# Understanding the Challenges of Increased Care Burden in Dementia Caregiving After Hospitalization

**DOI:** 10.1093/geroni/igaf122.1956

**Published:** 2025-12-31

**Authors:** Ashley Kuzmik, Lycia Tramujas Vasconcellos Neumann

## Abstract

For persons living with dementia and their care partners, the post-acute transition period presents unique challenges. Increased care needs, medical complexities, and limited formal support can heighten care partner burden, ultimately affecting care quality and patient outcomes. This symposium explores key factors contributing to care partner burden during post-hospital transitions, emphasizing the role of education, health literacy, dyadic characteristics, and burden’s impact on post-acute outcomes. The first presentation will examine how education and health literacy function as pathways to burden, specifically assessing the mediating role of health literacy on the relationship between education and burden. The second presentation will introduce a typology of dementia care dyads, identifying distinct care partner and care recipient groups and highlighting differences in burden across these groups. The third presentation will explore the association between care partner burden and negative post-acute outcomes, including emergency visits, hospital readmissions, falls, and injuries, with an emphasis on burden levels at discharge and changes over the post-discharge period. The symposium will conclude with a discussion on the implications of these findings for future research, interventions, and policy, with a focus on strategies to mitigate care partner burden and improve post-acute transitions in dementia care.

